# Couple interdependence impacts HIV-related health behaviours among pregnant couples in southwestern Kenya: a qualitative analysis

**DOI:** 10.7448/IAS.19.1.21224

**Published:** 2016-11-24

**Authors:** Anna Joy Rogers, Lillian Achiro, Elizabeth A Bukusi, Abigail M Hatcher, Zachary Kwena, Pamela L Musoke, Janet M Turan, Elly Weke, Lynae A Darbes

**Affiliations:** 1 Department of Health Care Organization and Policy, School of Public Health, University of Alabama at Birmingham, Birmingham, AL, USA; 2 Centre for Microbiology Research, Kenya Medical Research Institute, Nairobi, Kenya; 3 Department of Obstetrics and Gynaecology, University of Washington, Seattle, WA, USA; 4 Faculty of Health Sciences, University of the Witwatersrand, Johannesburg, South Africa; 5 Division of HIV/AIDS, Department of Medicine, University of California, San Francisco (UCSF), San Franscisco, CA, USA; 6 Center for Sexuality and Health Disparities, Department of Health Behaviour and Biological Sciences, University of Michigan School of Nursing, Ann Arbor, MI, USA

**Keywords:** HIV, health behaviour, pregnancy, qualitative, couples interventions, Africa

## Abstract

**Introduction:**

HIV infection is frequently transmitted within stable couple partnerships. In order to prevent HIV acquisition in HIV-negative couples, as well as improve coping in couples with an HIV-positive diagnosis, it has been suggested that interventions be aimed at strengthening couple relationships, in addition to addressing individual behaviours. However, little is known about factors that influence relationships to impact joint decision-making related to HIV.

**Methods:**

We conducted qualitative in-depth interviews with 40 pregnant women and 40 male partners in southwestern Kenya, an area of high HIV prevalence. Drawing from the interdependence model of communal coping and health behaviour change, we employed thematic analysis methods to analyze interview transcripts in Dedoose software with the aim of identifying key relationship factors that could contribute to the development of a couples-based intervention to improve health outcomes for pregnant women and their male partners.

**Results:**

In accordance with the interdependence model, we found that couples with greater relationship-centred motivations described jointly engaging in more health-enhancing behaviours, such as couples HIV testing, disclosure of HIV status, and cooperation to improve medication and clinic appointment adherence. These couples often had predisposing factors such as stronger communication skills and shared children, and were less likely to face potential challenges such as polygamous marriages, wife inheritance, living separately, or financial difficulties. For HIV-negative couples, joint decision-making helped them face the health threat of acquiring HIV together. For couples with an HIV-positive diagnosis, communal coping helped reduce risk of interspousal transmission and improve long-term health prospects. Conversely, participants felt that self-centred motivations led to more concurrent sexual partnerships, reduced relationship satisfaction, and mistrust. Couples who lacked interdependence were more likely to mention experiencing violence or relationship dissolution, or having difficulty coping with HIV-related stigma.

**Conclusions:**

We found that interdependence theory may provide key insights into health-related attitudes and behaviours adopted by pregnant couples. Interventions that invest in strengthening relationships, such as couple counselling during pregnancy, may improve adoption of beneficial HIV-related health behaviours. Future research should explore adaptation of existing evidence-based couple counselling interventions to local contexts, in order to address modifiable relationship characteristics that can increase interdependence and improve HIV-related health outcomes.

## Introduction

HIV is frequently transmitted within stable couple partnerships. In long-term intimate relationships, partners often exert substantial mutual influence, share health-related perceptions [1], and collaborate to improve health [2]. Therefore, it has been suggested that interventions aimed at improving couple relationships may help reduce HIV acquisition by HIV-negative couples and improve coping by couples in which one or both partners has an HIV-positive diagnosis [3].

Among sub-Saharan African (SSA) couples, the vast majority of HIV transmission occurs within enduring heterosexual partnerships [4, 5] and as many as half of all HIV-positive individuals are in an HIV-serodiscordant relationship [6–8]. Some factors associated with HIV transmission in this context include lower consistency of condom use [9], higher frequency of sexual contact [10], and unprotected sex out of a desire to conceive children [11]. Additionally, power imbalances, economic dependence, and traditional gender roles may contribute to intimate-partner violence, which has been associated with less ability to negotiate safer sex [12–14]. Conversely, unity and egalitarian decision-making may be protective against intimate-partner violence [15, 16]. This suggests that couple-focused HIV prevention efforts have the potential to greatly reduce transmission in this population.

In addition to preventing new infections, a dyadic approach may also help couples coping with an existing HIV diagnosis to improve HIV-related health and behaviour through the mechanism of social support. While family and community social support is associated with a wide range of health benefits, partner-specific social support has been shown to improve mutual emotional resilience, comprehension of information pertaining to the disease, and instrumental support in the form of financial, time, and travel assistance [17]. However, little is known about modifiable factors to strengthen couple relationships in order to prevent HIV acquisition, as well as improve communal coping – the cooperative process of appraising and addressing an individual or collective stressor [18] – with long-term HIV care and treatment in the SSA setting.

### Theoretical framework

We used the interdependence model of couple communal coping and behaviour change by Lewis *et al*. to guide our approach [19]. This interdependence model, adapted for our research context, suggests that couples may have one or more *predisposing factors* that influence whether they experience a *transformation of motivation* – a process whereby couples come to interpret health events as being meaningful to the relationship rather than simply for themselves as individuals. The interdependence model posits that relationship-centred motivation activates *communal coping* – a process in which couple members share an understanding about the health threat that they are facing and the courses of action required to manage the threat, and recognize the utility of a joint response. Ultimately, the ability to rely on each other for support impacts the likelihood of adopting and maintaining *health-enhancing behaviours*, thus influencing health outcomes. Interdependence is a key construct in this theoretical approach, and it refers to the ways in which interacting partners mutually influence each other's outcome [19]. This approach has been used previously in SSA settings [20, 21].

Our objective was to fill a gap in the literature by studying the impact of couple interdependence on the HIV-related health behaviours of pregnant couples in Kenya. The data from this study informed the development of a home-based couples HIV intervention for pregnant couples in Kenya.

## Methods

### Data collection

**Figure 1:**
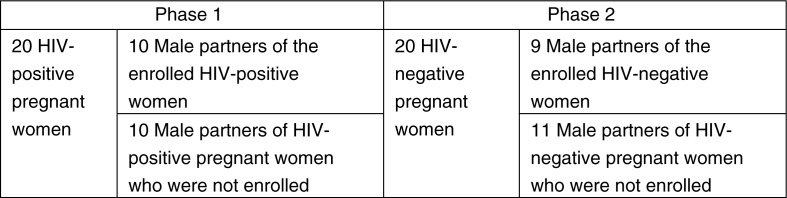
Pregnant women and male partners interviewed in each study phase.

We conducted formative research in two phases (Figure 1) through in-depth interviews with 40 pregnant women (half of whom were HIV-positive) and 40 male partners of pregnant women in rural Kenya. The first phase of the study was conducted in 2011 among HIV-positive pregnant women and male partners (half of whom were partners to the HIV-positive pregnant women who were enrolled, and half of whom were partners to unenrolled HIV-positive pregnant women) to explore how couples living with HIV would respond to a home-based couples HIV testing and counselling (CHTC) intervention. The second phase was part of a follow-up study in the same setting in 2014 among HIV-negative pregnant women and male partners of such women, in order to gain additional perspective and adapt the intervention design for all pregnant couples regardless of HIV status. As a result, although we had 40 pregnant women and 40 male partners, we did not have 40 male–female couple pairs, since half of each gender had a partner who was not enrolled in the study.

### Recruitment and eligibility

Participants were identified from six rural antenatal clinics affiliated with Family AIDS Care Education and Services (FACES) [22], a U.S. President's Emergency Plan for AIDS Relief (PEPFAR) funded initiative that supports health facilities in providing comprehensive HIV prevention, care, and treatment services. Lay healthcare workers who were trained in the research protocol recruited pregnant women who were 18 years of age or older and who had been offered HIV testing at antenatal clinics. For pregnant women who gave permission for researchers to contact their male partner, the male partners were contacted by researchers and invited for an interview.

### Interview guides and procedures

Qualitative interview guides for each phase were developed using interdependence theory in the context of the larger study about home-based CHTC and safe disclosure of HIV status within pregnant couples. The semi-structured interviews explored how couple relationship factors and interdependence may impact willingness to accept CHTC and adopt positive health behaviours. Participant demographics were collected using a brief standard questionnaire. Following signed informed consent, participants were interviewed by a gender-matched interviewer in a private room within the health facility for about one hour. They were reimbursed 400 Kenyan Shillings (roughly equivalent to US $5) for their time and any transportation expenses.

### Data analysis

Interviews were digitally recorded and transcribed verbatim in the local language (Kiswahili or Dhuluo) by professional transcriptionists, then translated into English. Transcripts were preliminarily coded and analyzed by a team of five researchers (AJR, LA, AMH, PLM, EW) in Dedoose (SocioCultural Research Consultants, LLC; Los Angeles, California) using a thematic analysis approach [23]. Through an iterative process involving a series of meetings by the research team, initial themes were identified and coded for based on the interdependence model. In a subsequent round of coding conducted by one researcher (AJR), fine codes were allowed to emerge inductively from the data and sub-themes identified. The final coding framework was discussed and approved by the whole team.

### Ethical approval

Ethical approval was given by the Kenya Medical Research Institute Ethical Review Committee, the University of Alabama at Birmingham Institutional Review Board, and the University of California at San Francisco Human Research Protection Program.

## Results

**Figure 2:**
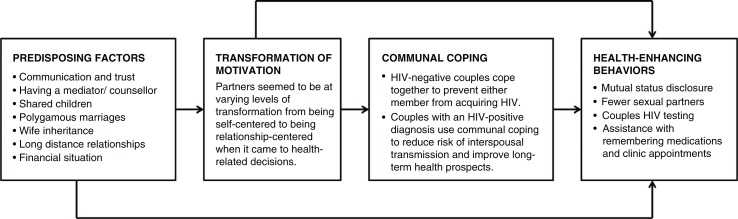
The interdependence model of communal coping and health behaviour change, adapted from Lewis *et al*. (19). Used with permission.

Participant characteristics are described in Table 1. The majority of women were early in their reproductive years and had completed a primary education. Men spanned the age spectrum and had a range of educational levels. Most participants were in a marital relationship (either monogamous or polygamous). A few women reported having a male partner, even though they listed their official status as being single or widowed. We utilized an adapted interdependence model [19] to explore how couple interdependence influenced the adoption of positive health behaviours by participants. Thus, the results of this study are presented in accordance with the four major themes – predisposing factors, transformation of motivation, communal coping, and health-enhancing behaviours – found in Lewis and colleagues’ interdependence model [19] (Figure 2). For the sake of brevity, most of the supporting participant quotes are presented in table format, but we included some in-text quotations to give our participant voices a presence in the main text.

**Table 1:** Interview participant characteristics

		Pregnant women		Male partners	
			
Characteristics	Total *N* (%)	HIV-positive *N*=20	HIV-negative *N*=20	Female partner is HIV-positive *N*=20	Female partner is HIV-negative *N*=20
Participant age (years)		---------------------------------*N* (%)---------------------------------			
18–24	26 (32.5)	8 (40)	10 (50)	3 (15)	5 (25)
25–34	32 (40.0)	10 (50)	10 (50)	7 (35)	5 (25)
35–44	12 (15.0)	2 (10)	0 (0)	7 (35)	3 (15)
≥45	10 (12.5)	0 (0)	0 (0)	3 (15)	7 (35)
Participant education					
Did not complete primary	23 (28.8)	5 (25)	6 (30)	9 (45)	3 (15)
Completed primary	29 (36.2)	11 (55)	5 (25)	4 (20)	9 (45)
Did not complete secondary	9 (11.3)	1 (5)	3 (15)	3 (25)	2 (10)
Completed secondary	13 (16.2)	2 (10)	3 (15)	4 (20)	4 (20)
Any college	6 (7.5)	1 (5)	3 (15)	0 (0)	2 (10)
Marital status					
Monogamous marriage	56 (70.0)	11 (55)	17 (85)	13 (65)	15 (75)
Polygamous marriage	20 (25.0)	5 (25)	3 (15)	7 (35)	5 (25)
Single	1 (1.2)	1 (5)	0 (0)	0 (0)	0 (0)
Widow	3 (3.8)	3 (15)	0 (0)	0 (0)	0 (0)
Currently living with spouse	65 (81)	10 (50)	18 (90)	18 (90)	19 (95)
Number of living children					
0	18 (22.5)	4 (20)	8 (40)	2 (10)	4 (20)
1	6 (7.5)	2 (10)	2 (10)	1 (5)	1 (5)
2	19 (23.7)	5 (25)	6 (30)	4 (20)	4 (20)
3 or more	37 (46.3)	9 (45)	4 (20)	13 (65)	11 (55)
Occupation^a^					
Agriculture	27 (34.6)	1 (5)	7 (35)	10 (50)	9 (45)
Small business/sales	12 (15.4)	5 (25)	4 (20)	2 (10)	1 (5)
Skilled or semi-skilled worker	26 (33.3)	3 (15)	5 (25)	8 (40)	10 (50)
Housewife	13 (16.7)	9 (45)	4 (20)	0 (0)	0 (0)
Pregnancy duration (weeks)^a^					
3–28 weeks	40 (51.2)	12 (60)	7 (35)	12 (60)	9 (45)
29–40 weeks	38 (48.8)	7 (35)	13 (65)	7 (35)	11 (55)

a There are missing values for two participants in each of these categories, so the total percentages have a denominator of 78 participants rather than 80.

### Predisposing factors

While some marital characteristics – such as communication, trust, having a mediator/counsellor, and sharing children – appeared to predispose couple members to feeling a greater connection to their partner (Table 2), several situations including polygamous marriages, the cultural tradition of wife inheritance, long-distance relationships, and financial difficulties presented challenges to couple interdependence (Table 3).

**Table 2:** Predisposing factors for couple interdependence

Characteristic	Exemplar text	Participant identifier
Communication and trust		
Mutual respect	“The respect that she has allows me to be free with her … If you take a keen look at your wife, you can find that she is a little sincere. This can encourage you to be free. You cannot understand each other or discuss anything if there is no respect between the two of you.”	Male partner #17, HIV-positive
Listening ability	“One can have an opinion but the other partner looks down upon it. Such things cannot encourage discussions in the family. In a family, one should be able to listen to what is being said so that they can all participate.”	Male partner #20, HIV-negative
Humour	“We discuss issues like recently we were discussing the number of children that we would want to have. Then he said five, but I said five is too many and asked him who would carry all those [pregnancies] … he just laughed and never said anything. We always joke with each other.”	Pregnant woman #19, HIV-negative
Spending time together	“[Getting more time as a couple] will bring peace to our family. You will never know what your partner thinks about the marriage unless you take your time to sit down with him or her for a conversation.”	Male partner #13, HIV-negative
Willingness to resolve conflicts	“Despite the fact that issues and quarrels must always be there in the house, we always sit and discuss … if it reaches a point that we can't communicate to each other because of small issues here and there then we will jeopardize our relationships [and] we may start being unfaithful to each other.”	Male partner #04, HIV-negative
Faithfulness in marriage	“The most important thing is faithfulness. I have never been unfaithful to my wife. But I do have a problem with the guys who want her… But I still trust her that there is no relationship between her and other men.”	Male partner #05, HIV-negative
Having a mediator/counsellor	“You know a woman and her husband cannot talk and agree on something that already got spoilt. But people's experiences from outside [the marital relationship] can make someone listen and this can bring peace in their house … someone from outside can teach you and you take these teachings and agree with each other.”	Pregnant woman #26, HIV-positive
Shared children	“[Marriage] is all about having children… You must be happy when the wife is pregnant. She is going to add another member of the family.”	Male partner #14, HIV-negative

#### Communication and trust

Couples appeared to be more predisposed to adopting healthy behaviours for the sake of the relationship when there was communication and trust between partners. To maintain a healthy household, participants described honesty and mutual respect as forming a foundation for the relationship. Participants felt that communication skills such as listening to their partner and incorporating humour into their discussions allowed for the strengthening of the relationship bond. Those who made a special effort to spend time together – whether at mealtimes or during activities of shared interest like walks – tended to report a willingness to refrain from concurrent sexual partnerships in order to protect their partner's health. Another key factor that predisposed couples to interdependence and marital faithfulness was a willingness to resolve conflicts.

Partners lacking in interdependence tended towards individual rather than joint decision-making. For example, one HIV-negative male who has concurrent sexual partners described not being able to trust that his wife is faithful: “This world is a very dangerous place to live in. Sometimes I can go for the [HIV] test without consulting her. I will do it secretly. She might also do it secretly” (Male partner #03, HIV-negative).

#### Having a mediator/counsellor

For couples who had difficulty overcoming disagreements or negotiating conflict, some participants felt that it may be helpful to have a counsellor from outside the relationship step in and help promote understanding between them. One pregnant woman described how having a counsellor could bring peace and negotiate agreement. A male partner shared a similar sentiment when he said that counsellors can “sense a problem during the discussion and help the couple” (Male partner #39, HIV-positive).

#### Shared children

For men, having children with their partners often helped them feel more connected to the relationship and, in turn, impacted their health-seeking decisions. Even if they did not attend antenatal clinic with their pregnant partners or involve themselves directly in the care of the children, men often expressed support for their wives and children engaging in clinical care by providing financially. For many of our male participants, children helped strengthen the relationship bond; as one male partner said: “Love comes first and then the child. These are the most important things in a relationship” (Male partner #07, HIV-negative). Women similarly expressed their desire for children, with a few stressing the necessity of getting pregnant. One woman who decided to stop using condoms said that she was “just in need of another child” (Pregnant woman #38, HIV-positive).

#### Polygamous marriages

Of the 80 participants in our sample, 20 were in a polygamous marriage. Some women described their struggle to maintain their HIV-negative status when they have little say in their husband's choice of other wives and no knowledge of the HIV status of their co-wives. In spite of the challenge, some partners successfully navigated the complexity of multiple sexual relationships within a household by being open with each other. As one HIV-positive man described:

I discussed with her the reality of us being in a polygamous marriage. That we are three people who are staying together and one of us has tested HIV-positive. This meant that we were all at risk; hence I had made a decision for us to go for the HIV test and if anyone tested positive it's better to know it and start early care and treatment than waiting to get more surprises. (Male partner #33, HIV-positive)

#### Wife inheritance

Among some communities in sub-Saharan Africa, including the Luo in southwestern Kenya, wife inheritance is a cultural practice whereby widows are inherited by a brother or close relative of their deceased husband [24, 25]. Several female participants mentioned wife inheritance as a challenge to maintaining one's relationship and HIV-negative status, because these women may have had husbands who died of AIDS and thus may also be infected.

#### Financial difficulties and long-distance relationships

When participants were unemployed or experiencing a financial shortage, many expressed that this put a strain on their relationship. For at least one male participant, the strain eventually led to his wife leaving him. Partnerships characterized by long-distance communication and weekly or monthly visits appeared to be prone to a lesser degree of relationship-centred health behaviours. Several pregnant women in such relationships described feeling abandoned or helpless should someone in the family fall sick. A few couples coped with this challenge by maintaining frequent phone contact, which allowed them to jointly make decisions for the benefit of the family, even though apart.

### Transformation of motivation

Our data suggest that transformation of motivation is a key construct when trying to understand the variation in health-related attitudes, behaviours, and outcomes among pregnant couples. Based partially on their unique constellation of predisposing factors, participants demonstrated varying degrees of motivation to address family health as a couple. They described this motivation through different means, including through their expressions of concern, affection, and fidelity. One indicator of relationship-centred motivations was the use of “we” language rather than “I” language when referring to health-seeking behaviours (Table 4). For example, one man described why he and his wife engage in health-seeking behaviours by saying: “I want us to take care of ourselves. If at all we still don't have [HIV], we need to take measures to continue protecting ourselves” (Male partner #14, HIV-negative). Participants expressed their unity by describing joint decision-making and consulting each other on matters pertaining to their health. Others expressed their transformation of motivation by disclosing their HIV-positive status for the sake of their partner's health.

**Table 3:** Predisposing factors that may hamper couple interdependence

Characteristic	Exemplar text	Participant identifier
Lack of communication and trust		
Dishonesty	“You should not live lying to each other. Sometimes someone lies to the other, they are sick and the other person is healthy. So this other person will bring death to the other here in their house because there is no openness and the healthy person will contract the disease. If you are open with each other you can find a way of preventing this.”	Pregnant woman #27, HIV-positive
Mistrust	“If there is mistrust in the house, there will be no communication in that house because you cannot share with me something and you do not trust me and myself I will do the same to you if you do not trust me.”	Male partner #23, HIV-positive
Polygamous marriages	“When the HIV virus strikes, for one to know that one of us is sick and the other is not becomes hard, since he marries every now and then, yet he doesn't want to reveal his status to me. When you ask him if he has gone for a test with these women he says … I should stay away. At the end of it, I am left staring since I can't do anything about it …”	Pregnant woman #06, HIV-negative
Wife inheritance	“There are so many women who have lost their husbands so you find them coming to your husband for companionship purposes. You find that this woman tries all ways and means until she gets your husband to inherit her, yet you do not know the nature of the disease or ailment that led to her husband's death.”	Pregnant woman #01, HIV-negative
Financial difficulties	“She realized that my income had depreciated. She decided to run away with the money she had collected from the business. She left me with a child and I found it difficult to stay alone with the child.”	Male partner #07, HIV-negative
Long-distance relationships	“Currently, life is difficult. I am very far from my wife. The child might become sick … She is like a single parent in the house … I therefore think we need to be together … She might wonder whether I have rejected her or not … So being close to her is good because you can help her in a way or another.”	Male partner #03, HIV-negative

In contrast, a minority of participants described circumstances that demonstrated lack of couple motivation. For example, one HIV-positive woman unilaterally chose to initiate antiretroviral therapy although her husband, who lives separately from her and openly engages in sexual relationships with other partners, remains in denial about their HIV-positive status: “I would be very happy if he knows about my health because he is the one who infected me [with HIV], but if he has refused then I feel that I should rescue my own life if he is not interested” (Pregnant woman #21, HIV-positive).

Participants also differed in the time point in which they seemed to transition from being more self-focused to being more relationship-focused in terms of their health behaviours. A minority of participants were relationship-focused from the beginning of their relationship, choosing to engage in CHTC before initiating sexual intimacy. Other participants appeared to have experienced a more gradual process of motivation transformation, as they changed their behaviour from engaging in concurrent sexual partnerships to being faithful to their current partner.

Pregnancy seemed to be a time period that positively influenced the transformation process, as the predisposing factor of having children together suggests. Many couples recognized the impact that being infected with HIV could have on their children: in addition to concerns about transmitting HIV perinatally, several expressed fear that not taking antiretroviral therapy would leave them too weak to take care of their children or even leave their children orphaned. Pregnancy also signalled the need for health checks at antenatal clinic, which spurred discussions of health and presented women with the option of testing for HIV, while also encouraging women to bring their spouses to the clinic. For some couples, pregnancy influenced them to have a positive shift in attitude towards their partners. As one man stated, “The truth is that since the pregnancy, we have never quarreled neither have I changed my mind or turned my back at her. At the same time I haven't seen her hate me” (Male partner #04, HIV-negative). For a few couples, pregnancy may have precipitated a negative attitude or contributed to a strain in the relationship. One woman in particular reported an increased frequency of physical abuse throughout her gestational period. Although there were some exceptions, pregnancy appeared to exert a significant influence on the unity and mutuality of the relationship.

While it was possible to identify whether couples had experienced some degree of transformation of motivation, it was difficult to determine the specific process by which this occurred. This shift was often expressed in the overarching narrative of the interview, rather than in clear demonstrative quotes. Similarly, it was not always possible to pinpoint a time at which the “transformation” occurred. From many of the stories, it appeared that this process may have occurred gradually over the course of their relationship.

### Communal coping

For couples in which both partners are HIV-negative, communal coping is essential to prevent either member from acquiring HIV. For couples who are dealing with an HIV diagnosis, communal coping may help to reduce risk of interspousal transmission and improve long-term health prospects (Table 5).

**Table 4:** Transformation of motivation

Characteristic	Exemplar text	Participant identifier
Indicators of relationship-centred motivations		
Use of “we” rather than “I” language	“I want us to take care of ourselves. If at all we still don't have [HIV], we need to take measures to continue protecting ourselves.”	Male partner #14, HIV-negative
Disclosing for sake of partner's health	“… [If] I know my status and I am on drugs and she is not then I will be doing her more harm. If we make love without protection she will be more disadvantaged because I am on drugs and she is not.”	Male partner #31, HIV-positive
Time of transformation of motivation		
Prior to marriage	“We however didn't go straight into marriage because we wanted to go for the [HIV] test first … in case the lady was sick, then she has infected me. I would have also infected her in case I was sick.”	Male partner #02, HIV-negative
During pregnancy	“The truth is that since the pregnancy, we have never quarrelled neither have I changed my mind or turned my back at her. At the same time I haven't see her hate me.” (Male partner #04, HIV-negative)	Pregnant woman #01, HIV-negative

**Table 5:** Communal coping to improve health-enhancing behaviours

Characteristic	Exemplar text	Participant identifier
Communal coping within HIV-negative couples		
Couples HIV testing and counselling (CHTC) Abstaining from concurrent sexual relationships	“[Couples] should get tested together to know their HIV status. Going for HIV test can make one to have the fear of engaging in extra marital affairs because once they are HIV-negative, they would want to protect themselves. There are very many beautiful ladies who are very tempting outside here but knowing my HIV status is what has been keeping me in check.”	Male partner #18, HIV-negative
Preparing mentally for the HIV test	“I first discussed with her in the house before we left for the clinic that we were going to be tested together for HIV to know our status early enough so that in case we are HIV-positive then we can seek help. I told her not to be fearful but to be confident as much as we didn't know what the results would be.”	Male partner #04, HIV-negative
Communal coping within couples that have an HIV-positive diagnosis		
Resolve negative emotions	“I felt bad because… I felt that we were still too young and could not possibly be having HIV virus, but he tried talking to me until I accepted the facts, he also told me that he got courage after he was counselled from the hospital … I became courageous. When I enrolled for the ARVs [antiretroviral medications] I did not feel anything.”	Pregnant woman #36, HIV-positive
Cope with stigma	“I told her that in today's life, everyone is either infected or affected hence it is not strange that one is enrolled on care and advised her to go the to the hospital and take medicine.”	Male partner #21, HIV-positive
Medication adherence and clinic appointment reminders	“If they talk about [HIV] in the right and peaceful manner in their house, that is one thing that can unite them in their house, one of the good things about talking to your wife about your HIV status that men can see is that these women can remind them to swallow their medicine and also the date of attending adherence, it becomes their family responsibility.”	Male partner #31, HIV-positive
Avoid suicidal ideation	“If you don't understand each other then it becomes disadvantageous because everyone thinks of their own things. Someone might feel that since they have the disease then they should just die or do something that is not right and this will endanger their health.”	Pregnant woman #24, HIV-positive
Avoid violence and separation	“My sister found a man and got married [to him] without knowing that he was on medication [for HIV]. They stayed [in a relationship] for a while, gave birth to a child and decided to have some tests done. When she came back to tell the husband [of her HIV-positive status], he was very harsh and chased her away. She didn't take the husband seriously. He came back home very drunk, took a machete and chased her away.”	Pregnant woman #34, HIV-positive

#### HIV-negative couples

HIV-negative partners discussed some of the actions that they took together to prevent themselves from acquiring HIV. Couples who appear to have experienced a transformation of motivation were willing to accept CHTC, a process whereby both partners determine their HIV status and mutually disclose to each other. Some couples who made HIV testing a regular strategy felt that it helped them maintain mutual trust and abstain from concurrent sexual relationships. The vulnerability inherent in testing and disclosure meant that couples had to entrust their partners with their HIV status. For example, some participants even described helping each other prepare mentally for the HIV test as a means of communal coping, while others felt that after getting a few HIV tests, they were no longer nervous about the outcome as they had gained confidence in their partners.

In contrast to couples testing together, one HIV-negative participant stated that she had gone for HIV testing alone over ten times because her husband had “developed a behaviour of sleeping around with ladies from the bar” (Pregnant woman #06, HIV-negative).

#### Couples with an HIV-positive diagnosis

For couples who were already aware of an HIV diagnosis in at least one partner, communal coping took on several forms. Disclosure, which requires trusting the individual that you disclose to, can be an indication that partners have experienced a transformation of motivation. Some participants described the internal struggle that they experienced when trying to disclose, sometimes taking a few days or weeks to gather the courage. Other participants disclosed early, describing their relationships as open and honest. In either case, the other partner's health was always of concern.

Communal coping also helped couples deal with the news of an HIV diagnosis, such as through processing negative emotions and resolving fear. Mutual encouragement helped couples combat perceived or experienced stigma, remember to take medications, and adhere to clinic appointments. Many participants in interdependent relationships described how adhering to HIV care and treatment regimens became a family responsibility.

Conversely, if partner support was absent in the wake of disclosure, at least one participant described that the pressure, fear, and stigma of an HIV diagnosis may lead some people to have suicidal ideation or exhibit other harmful behaviour. Participants who were unable to cope with their HIV status together often experienced intimate-partner violence or even separation from their partners. These individuals often had a weaker foundation to their relationship due to predisposing factors such as poor communication skills, possibly compounded by other issues such as financial strain.

### Health-enhancing behaviours

As can be seen in the data presented above, communal coping appeared to lead to several health-enhancing behaviours, such as mutual disclosure, regular HIV testing, getting tested together as a couple, fewer sexual partners, and help with remembering medications and appointments. Conversely, participants with self-centred motivations often continued to have multiple sexual partners, avoid testing, prefer to keep their HIV status a secret, and be vulnerable to stigma, violence, and dissolution of their relationships.

## Discussion

This qualitative investigation of pregnant women and male partners, conducted in a region of Kenya with high HIV prevalence, was designed to utilize an adaptation of the interdependence model developed by Lewis et al. [19], as a lens through which to explore how couple interdependence impacts the adoption of beneficial HIV-related health behaviours.

We identified several key findings that are in accordance with the interdependence model. First, strong communication skills and mutual trust appeared to be key factors that predisposed a couple to greater interdependence. While research has focused on couple communication in the context of negotiating safer sex [26–28] and disclosing HIV status [29, 30], less is known about how openness within a relationship may impact a broader array of HIV-related behaviours. Our data suggest that a host of modifiable communication skills such as honesty, listening, and a willingness to resolve conflicts may increase the ability of a couple to see their health as a mutual responsibility, rather than the prerogative of one individual. These characteristics may be especially important for couples seeking to overcome the unique challenges posed by polygamous marriages, wife inheritance, long-distance relationships, and financial difficulties. Couples found the intervention of a counsellor to be acceptable and even desirable to aid in communication, since a mediator from outside the relationship may be respected by both parties. Counselling may additionally protect against relationship dissolution, particularly in serodiscordant couples [31]. This is in line with expert calls for dyadic interventions to not only focus on safer sex behaviours and disclosure [3, 32] but also include relationship-skills building [33].

Second, we identified that pregnancy may be a key time for some couples to shift from having more self-centred motivations to being more relationship-centred in their approach towards health and wellness. Other research from the region suggests that while couples prefer large families, having an HIV diagnosis in one couple member may lead to more joint decision-making regarding childbearing, in particular, weighing the risks of vertically transmitting HIV to offspring with a desire to have enough descendants or “replace” children who had passed away due to HIV [34, 35]. This finding was strengthened by our data suggesting that sharing children predisposed couples to being interdependent, and research from the literature showing that in the SSA context, children are an important contributor to relationship stability [31].

Third, we found that communal coping, as exhibited by mutual disclosure, CHTC, and assistance with medications and appointments, was an indicator of overall relationship strength. It had a significant impact on the ability of the couple to navigate the challenges of avoiding HIV acquisition if they were both HIV-negative, preventing HIV transmission if they were serodiscordant, and living positively with HIV. Conversely, a lack of couple interdependence and communal coping was sometimes associated with stigma and violence, a phenomenon that is reported on extensively in the literature [36, 37].

The current study has several strengths including the presence of both HIV-positive and HIV-negative participants, allowing us to assess the differential impact of HIV on interdependence and communal coping. Our inclusion of male partners’ voices, which have only recently begun to be included in many studies, contributed to the richness of the data.

The limitations of this qualitative cross-sectional study include the inability to infer causal relationships between predisposing factors and health-related behaviours and outcomes. Additionally, because our interviews were conducted at a single point in time, it was not possible for us to make conclusions about the longitudinal relationship strength of our participant couples. Another limitation is that since male partners were only contacted for an interview if the pregnant woman was comfortable with him being contacted to discuss topics related to HIV, our sample is likely biased to include more supportive male partners, as compared to the general population of pregnant couples in this setting. Finally, more work is needed to explore the applicability of this theory to the larger SSA context [20].

## Conclusions

In conclusion, our data suggest that theoretical constructs defined by interdependence theory – specifically predisposing factors of a couple, transformation of motivation, and communal coping – may provide key insights when trying to understand and influence health-related attitudes and behaviours adopted by pregnant couples in SSA. Our data also corroborate expert calls for HIV interventions to strengthen couple relationships rather than solely focusing on modifying individual behaviours that increase risk of HIV transmission. One such intervention could be improving access to counselling services to improve communication skills, which our study suggests may predispose them to jointly adopt healthier behaviours. Future research should explore adaptation of existing evidence-based couple counselling interventions, such as Pettifor and colleagues’ adaptation of Project Connect [38–40], to local contexts in order to address modifiable relationship characteristics that can increase interdependence and improve HIV-related health outcomes. Programmes that focus on strengthening couple relationships during pregnancy may have an enduring positive influence on the general and HIV-related health of pregnant women, their male partners, and their children.
